# Fibroblast growth factor 23 is independently associated with renal magnesium handling in patients with chronic kidney disease

**DOI:** 10.3389/fendo.2022.1046392

**Published:** 2023-01-09

**Authors:** Teodora V. Grigore, Malou Zuidscherwoude, Anna Witasp, Peter Barany, Annika Wernerson, Annette Bruchfeld, Hong Xu, Hannes Olauson, Joost Hoenderop

**Affiliations:** ^1^ Radboud Institute for Molecular Life Sciences, Department of Physiology, Radboudumc, Nijmegen, Netherlands; ^2^ Karolinska Institutet, Division of Renal Medicine, Department of Clinical Science, Intervention and Technology, Stockholm, Sweden; ^3^ Linköpings universitet Hälsouniversitetet, Department of Health, Medicine and Caring Sciences, Linköping, Sweden; ^4^ Karolinska Institutet, Division of Clinical Geriatrics, Department of Neurobiology, Department of Care Sciences and Society, Stockholm, Sweden

**Keywords:** FGF23, FEMg, CKD, Klotho, phosphate

## Abstract

**Background:**

Disturbances in magnesium homeostasis are common in patients with chronic kidney disease (CKD) and are associated with increased mortality. The kidney is a key organ in maintaining normal serum magnesium concentrations. To this end, fractional excretion of magnesium (FEMg) increases as renal function declines. Despite recent progress, the hormonal regulation of renal magnesium handling is incompletely understood. Fibroblast Growth Factor 23 (FGF23) is a phosphaturic hormone that has been linked to renal magnesium handling. However, it has not yet been reported whether FGF23 is associated with renal magnesium handling in CKD patients.

**Methods:**

The associations between plasma FGF23 levels, plasma and urine magnesium concentrations and FEMg was investigated in a cross-sectional cohort of 198 non-dialysis CKD patients undergoing renal biopsy.

**Results:**

FGF23 was significantly correlated with FEMg (Pearson’s correlation coefficient = 0.37, p<0.001) and urinary magnesium (-0.14, p=0.04), but not with plasma magnesium. The association between FGF23 and FEMg remained significant after adjusting for potential confounders, including estimated glomerular filtration rate (eGFR), parathyroid hormone and 25-hydroxyvitamin D.

**Conclusions:**

We report that plasma FGF23 is independently associated with measures of renal magnesium handling in a cohort of non-dialysis CKD patients. A potential causal relationship should be investigated in future studies.

## 1 Introduction

Magnesium ions (Mg^2+^) are essential in several biological processes such as cell signaling, energy metabolism, growth and proliferation. Normomagnesemia is maintained between 0.7 and 1.1 mmol/L through an elaborate coordination between the kidney, bone and intestine. Renal excretion of Mg^2+^ is a controlled process (1) fine-tuned in the distal convoluted tubule (DCT). In the DCT, regulated Mg^2+^ reabsorption occurs through the transient receptor potential melastatin (TRPM) 6 (2). 10-25% filtered Mg^2+^ is reabsorbed in the proximal tubule and 50-70% is reabsorbed in the thick ascending limb of Henle’s loop. Fine-tuning of Mg^2+^ reabsorption occurs in the DCT (10%) *via* an active transcellular process (1). The Mg^2+^ handling in the kidney is influenced by several factors. Generally, peptide hormones, such as parathyroid hormone (PTH) or vasopressin, increase the Mg^2+^ transport (3). Interestingly, vitamin D does not influence, in physiological conditions, the Mg^2+^ homeostasis, yet rodents treated with vitamin D showed an increase in serum levels of Mg^2+^ and a decrease in renal Mg^2+^ excretion (4). Electrolytes play an important role in the transport of Mg^2+^. Calcium (Ca^2+^) and Mg^2+^ ions have been shown to activate Calcium Sensing Receptor (CaSR) and regulate paracellular Mg^2+^ transport (5), while potassium (K^+^) indirectly influences the Mg^2+^ transport in the DCT through the voltage-gated channel Kv1.1, which provides an electrical driving force for transcellular Mg^2+^ transport (6, 7). Nevertheless, the exact regulation of renal Mg^2+^ handling remains largely unkown. Mutations in the *TRPM6* gene results in primary hypomagnesemia with secondary hypocalcemia (PHSH, OMIM# 602014), an autosomal recessive condition characterized by abnormally low serum Mg^2+^. However, the precise hormonal regulation of TRPM6 in health and in disease has not yet been characterized (8).

Chronic kidney disease (CKD) affects approximately 10% of the population globally and is associated with increased cardiovascular morbidity and mortality (9). Vascular calcification is a key mechanism behind the increased cardiovascular risk associated with CKD (10, 11). In CKD patients the fractional excretion of Mg^2+^ (FEMg) gradually increases to compensate for the reduced number of functioning nephrons (12). In patients with end stage renal disease (ESRD) the adaptive mechanisms are no longer sufficient to fully compensate for the loss of glomerular filtration capacity, which can lead to hypermagnesemia (13). Also, hypomagnesemia is commonly observed in CKD patients due to reduced intestinal uptake or increased renal losses caused by use of medication such as diuretics and proton-pump inhibitors (14, 15), comorbidities including diabetes and hypertension, or low dietary Mg^2+^ intake (1).

Both hyper- and hypomagnesemia have been linked to cardiovascular disease and higher overall mortality in patients with CKD (16, 17). Mechanistically, Mg^2+^ has been demonstrated to inhibit phosphate-induced vascular calcification, and Mg^2+^ supplementation or Mg^2+^-based therapies have shown promise in reducing cardiovascular disease in CKD patients (18–22). A better understanding of the hormonal regulation of renal Mg^2+^ handling, especially in the setting of reduced renal function, is therefore of outmost clinical importance.

Fibroblast Growth Factor 23 (FGF23) is a bone-derived hormone that reduces the apical abundance of the sodium-phosphate co-transporters NPT2A and NPT2C in the proximal tubule, thereby lowering phosphate (Pi) reabsorption and increasing urinary Pi excretion. FGF23 concentrations increase dramatically in CKD patients, likely as an adaptive mechanism to counteract Pi retention (23–25). FGF23 plays an active role in the inhibition of renal 1,25-dihydroxyvitamin D (1,25(OH)_2_D) synthesis by increasing the expression of the calcitriol-inactivating enzyme 24-hydroxylase, whereas PTH stimulates the 25-hydroxyvitamin D-1α-hydroxylase, which generates 1,25(OH)_2_D (26). FGF23 and 1,25(OH)_2_D regulate each other through a feedback loop, as 1,25(OH)_2_D stimulates FGF23 synthesis in osteoblasts through the presence of a vitamin D response element in the FGF23 promoter (27). Animal studies show that FGF23 inhibits PTH secretion by activating the MAPK pathway, which decreases PTH expression and secretion (28, 29). This mechanism may not be important in humans, as CKD patients display a simultaneous increase in PTH and FGF23 levels (30–32).

Many epidemiological studies have linked increased FGF23 concentrations to worse clinical outcomes, including cardiovascular morbidity and mortality (33, 34), although the evidence of a causal relationship from prospective trials is still lacking.

In addition to regulating Pi balance, FGF23 and its co-receptor Klotho have been linked to renal handling of other electrolytes, including Ca^2+^, sodium (Na^+^) and K^+^ (35–37). There are also some indications that FGF23 may be involved in controlling renal Mg^2+^ reabsorption. First, mice with diet-induced hypomagnesemia have increased serum FGF23 levels and reduced renal Klotho expression (38, 39), suggesting that dietary Mg^2+^ can influence the regulation of the FGF23-Klotho axis. Similarly, low plasma Mg^2+^ concentrations are associated with high plasma FGF23 concentrations and high mortality rates in azotemic cats with CKD (40). Second, a negative association between serum FGF23 and Mg^2+^ was observed in patients undergoing hemodialysis, supporting a possible interrelationship (41). Third, Mg^2+^ supplementation was recently reported to reduce vascular calcification in hyperphosphatemic Klotho-deficient mice with high FGF23 concentrations (8).

Herein we set out to test whether FGF23 could be involved in renal Mg^2+^ handling in patients with non-dialysis CKD, and particularly we hypothesize that FGF23 and FEMg are positively associated.

## 2 Materials and methods

### 2.1 Study design and patient inclusion

For the present study we used a cohort of non-dialysis patients with CKD stage 1-5 undergoing kidney biopsy at the Karolinska University Hospital between 2011 and 2017 (KaroKidney – karokidney.org) (23). All patients in which plasma and spot urine Mg^2+^ were available were included in the analysis. The study received ethical approval (Ethical Review Board, Stockholm, Sweden, DNR 2010/579-31), and informed consent was obtained from all participants.

### 2.2 Exposure, outcome and covariates

Prior to the kidney biopsy, blood and urine samples were collected and directly analyzed or frozen and stored at -80°C for later biochemical measurements. Intact FGF23 was measured in plasma using a second-generation enzyme-linked immunosorbent assay (ELISA) (Immutopics, San Clemente, CA, USA). Plasma (normal values equal to 0.7-1.1 mmol/L) and urine Mg^2+^ concentrations were measured using a colorimetric assay (Roche/Hitachi, Tokyo, Japan) according to the manufacturer’s protocol. Absorbance was measured at a 600 nm wavelength on a microplate spectrophotometer (Bio-Rad Laboratories, CA, USA). All Mg^2+^ measurements were carried out in triplicate. The FEMg (normal range defined as FEMg under 4%) was calculated using the formula [(U_Mg_^2+^ x P_Crea_)/(U_Crea_ x P_Mg_^2+^ x 0.7)] x 100 (where U = urinary, P = plasma, Mg^2+^ = ionized magnesium, Crea = creatinine) (42). Soluble Klotho and aldosterone were analyzed in serum using ELISA methods (IBL International, Germany and DRG Diagnostics, Germany, respectively). Estimated eGFR was calculated using plasma Cystatin C values.

### 2.3 Statistical analysis

Data were expressed as mean ± standard deviation (SD) or median with interquartile range (IQR) for continuous variables and percentage of total for categorical variables. Plasma FGF23, Klotho, 25-hydroxyvitamin D, PTH, aldosterone, fractional excretion of Ca^2+^ (FECa), FEMg, fraction excretion of K^+^ (FEK), plasma Pi, Ca^2+^, creatinine, and urinary Ca^2+^, Mg^2+^, Pi and creatinine were log_10_ transformed when used in models, to improve their distribution towards normal. Missing data were imputed by using Predictive Mean Matching with 15 iterations and can be found in **Supplementary Table 1**.

The cohort was stratified into quartiles, based on the plasma levels of FGF23, and the quartiles were tested for group comparisons using one-way ANOVA and Tukey’s *post-hoc* test to correct for multiple comparisons.

Pearson’s correlation was used to explore the relationships between FGF23, FEMg, eGFR, age, sex, aldosterone, PTH, Klotho, 25-hydroxyvitamin D, FEK, fractional excretion of Pi (FEPi), FECa, plasma Na^+^, K^+^, Ca^2+^, Pi, Mg^2+^, urinary Ca^2+^, and Pi concentrations. All data were compiled in a correlation matrix with all covariates included.

Univariate linear regression models were performed to investigate the effect of FGF23 on FEMg values. Data were expressed as regression coefficient (β), 95% confidence intervals (CI), adjusted R^2^ and *p*-value. Multivariate general linear regression models were run to investigate the effect of FGF23 on FEMg. The first model (model 1) consisted of age (years), sex and eGFR (mL/min/1.73 m^2^). The second model (model 2) included the same parameters as model 1 plus Klotho (pg/mL), 25-hydroxyvitamin D (nmol/L), and PTH (ng/L). The third model (model 3) included model 1 and medication (yes/no): loop diuretics, thiazide, PPI, and beta blockers. The fourth model (model 4) included model 2 and model 3, and the fifth model (model 5) included model 4 and comorbidities (yes/no): hypertension and diabetes. The sixth model (model 6) included model 2 and FECa, while the seventh model (model 7) included model 1 and FECa, FEK and FEPi.

The cohort was divided into quartiles of FGF23 concentrations and quartiles of eGFR, to investigate if the log_10_FEMg associations are consistent even if the cohort is divided and we see the same trend. Linear regression analysis was used to show the associations.

Differences with a *p*-value of <0.05 were considered statistically significant. Statistical analyses were performed using statistical software SPSS for Windows, version 25, release 25.0.0.1, 64-bit edition and GraphPad Prism 8 for macOS, version 8.4.3 (471).

## 3 Results

### 3.1 Baseline characteristics of study population

The study cohort included a total of 198 CKD patients. Clinical characteristics and relevant laboratory results for the full cohort and FGF23 strata are reported in **Table 1**. Of the 198 patients, 62.7% were male, the mean age was 50.5 ( ± 17.0) years, the median for FGF23 was 81 (58 – 154) pg/mL and the mean eGFR was 48.7 ( ± 22.3) mL/min/1.73m^2^. Of the patients, 13.1% were hypomagnesemic and 57.1% presented FEMg values higher than the normal range. Of note, FEMg, FEPi and FEK increased over quartiles of FGF23. All plasma and urine electrolyte values are shown in **Supplementary Figure 1**.

**Table 1:** Baseline characteristics of patients in the CKD cohort (N=198) and of each FGF23 strata.

	Full cohort	Quartile 1	Quartile 2	Quartile 3	Quartile 4	p value for trend
N	**198**	**48**	**52**	**48**	**50**	
**Demographics**						
Age (years), mean ± SD	50.5 ( ± 17.0)	48.0 ( ± 17.4)	48.6 ( ± 16.1)	49.8 ( ± 18.2)	55.6 ( ± 15.7)	0.10
Sex (male), %	62.7	54.8	64.6	58.5	71.7	
**Laboratory analyses**						
FGF23 (pg/mL), median (IQR)	81.0 (58.0-154.0)	44.0 (33.2-53.0)	65.2 (61.1-71.0)	107.0 (94.3-125.0)	243.0 (186.5-373.5)	<0.0001
GFR Cystatin C (mL/min/1.73 m^2^), mean ± SD	48.7 ( ± 22.3)	61.9 ( ± 19.1)	57.3 ( ± 18.3)	48.3 ( ± 20.8)	27.4 ( ± 13.2)	<0.0001
Klotho (pg/mL), median (IQR)	640.5 (536.2-780.5)	748.5 (575.7-932.5)	653.5 (560.2-737.0)	637.5 (553.0-732.5)	571.0 (487.5-745.2)	0.005
PTH (ng/L), median (IQR)	9.1 (4.6-33.0)	6.4 (3.8-29.0)	9.6 (5.2-37.0)	6.7 (4.7-13.0)	11.5 (5.6-33.2)	0.05
25-hydroxyvitamin D (nmol/L), median (IQR)	37.5 (24.0-53.2)	39.5 (33.2-54.5)	39.0 (28.2-57.2)	31.0 (18.2-49.7)	35.5 (25.5-49.0)	0.78
Plasma creatinine (µmol/L), median (IQR)	121.5 (82.7-182.2)	82.0 (69.2-117.2)	100.0 (78.2-132.2)	123.0 (98.0-186.0)	209.0 (157.0-300.7)	<0.0001
Urinary creatinine (mmol/L), median (IQR)	6.4 (4.9-9.4)	6.8 (5.3-11.1)	6.7 (5.4-9.4)	6.3 (5.0-8.3)	5.6 (4.6-8.3)	0.55
Plasma Mg^2+^ (mmol/L), mean ± SD	0.8 ( ± 0.1)	0.8 ( ± 0.1)	0.8 ( ± 0.1)	0.8 ( ± 0.1)	0.8 ( ± 0.1)	0.81
Urinary Mg^2+^ (mmol/L), median (IQR)	2.0 (1.1-3.0)	2.3 (1.4-3.0)	2.2 (1.3-3.5)	1.9 (1.1-2.8)	1.7 (1.1-2.3)	0.13
FEMg (%), median (IQR)	4.8 (2.5-8.1)	3.2 (1.9-4.7)	4.1 (2.3-8.6)	5.0 (3.3-7.5)	7.5 (4.7-13.1)	<0.0001
Plasma Ca^2+^ (mmol/L), median (IQR)	2.2 (2.1-2.3)	2.2 (2.1-2.3)	2.2 (2.2-2.3)	2.2 (2.1-2.3)	2.2 (2.1-2.3)	0.56
Urinary Ca^2+^ (mmol/L), median (IQR)	0.5 (0.1-1.2)	0.9 (0.3-1.7)	0.5 (0.2-1.4)	0.4 (0.1-1.2)	0.2 (0.1-0.6)	0.002
FECa (%), median (IQR)	0.4 (0.1-0.7)	0.4 (0.1-0.7)	0.3 (0.1-0.9)	0.3 (0.1-0.7)	0.4 (0.1-1.1)	0.24
Plasma PO_4_^3-^ (mmol/L), median (IQR)	1.1 (0.9-1.3)	1.0 (0.8-1.1)	1.0 (0.8-1.2)	1.2 (1.0-1.3)	1.3 (1.1-1.5)	<0.0001
Urinary PO_4_^3-^ (mmol/L), median (IQR)	14.0 (8.6-20.0)	14.0 (7.8-22.9)	15.4 (10.5-20.6)	13.0 (7.4-19.4)	13.4 (9.4-16.3)	0.65
FEPi (%), mean ± SD	23.3 ( ± 14.3)	16.3 ( ± 10.8)	19.8 ( ± 12.4)	21.2 ( ± 11.1)	35.2 ( ± 14.8)	<0.0001
Plasma K^+^ (mmol/L), mean ± SD	4.0 ( ± 0.46)	3.8 ( ± 0.3)	3.9 ( ± 0.2)	4.1 ( ± 0.5)	4.2 ( ± 0.5)	<0.0001
FEK (%), median (IQR)	15.7 (10.1-22.5)	12.8 (7.4-18.5)	14.3 (10.1-19.4)	15.2 (10.2-21.6)	26.0 (16.3-34.9)	<0.0001
**Medication, (%)**						
Betablockers	34.8	24.4	23.1	43.2	46.9	
Loop diuretics	35.3	19.5	23.1	38.6	57.1	
Thiazides	4.9	4.9	5.8	4.7	4.1	
Ca^2+^-based PO_4_^3-^ binders	10.3	12.2	9.6	6.8	12.2	
Non-Ca^2+^-based PO_4_^3-^ binders	4.9	2.4	1.9	0	14.3	
PPI	17.4	22.0	13.5	13.6	20.4	
**Comorbidities, (%)**						
Hypertension	45.5	33.3	40.4	47.9	60.0	
T1D	1.5	2.1	0	43.2	4.0	
T2D	12.1	10.4	9.6	0	18.0	

Data reported as mean and SD for variables with normal distribution, and as median and IQR for skewed variables.

### 3.2 Associations between FGF23, covariates and renal magnesium handling

**Figure 1:**
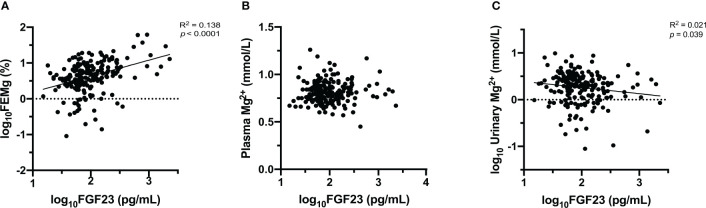
Association between FGF23 and Mg^2+^ [FEMg **(A)**, plasma Mg^2+^ **(B)**, urinary Mg^2+^ **(C)**]. Data are the outcomes of simple linear regression analysis.

Initially, the relationships between FGF23 and plasma Mg^2+^, urinary Mg^2+^ concentrations, and FEMg were explored using Pearson’s correlation (as shown in **Figure 1** and **Supplementary Table 2**). This analysis showed a significant correlation between FGF23, FEMg and urinary Mg^2+^, but no significant correlation with plasma Mg^2+^ concentration. Furthermore, plasma FGF23 was negatively correlated with plasma Klotho (r=-0.281, *p*<0.001), and positively correlated with FEPi (r=0.527, *p*<0.001) (shown in **Figure 1** and **Supplementary Table 2**).

Next, Pearson’s correlation was used to assess the relationship between FEMg and several clinical characteristics. The bivariate analysis demonstrated a negative correlation between FEMg and eGFR (r=-0.397, *p*<0.001) and an inversed correlation with plasma Klotho (r=-0.261, *p*<0.001), as seen in **Figure 1** and **Supplementary Table 2**. Further, FEMg was positively and moderately correlated with FEPi (r=0.394, *p*<0.001) and plasma Pi (r=0.229, *p*=0.001) (shown in **Figure 1** and **Supplementary Table 2**).

### 3.3 Univariate and multivariate analyses between FGF23 and renal magnesium handling

**Figure 2:**
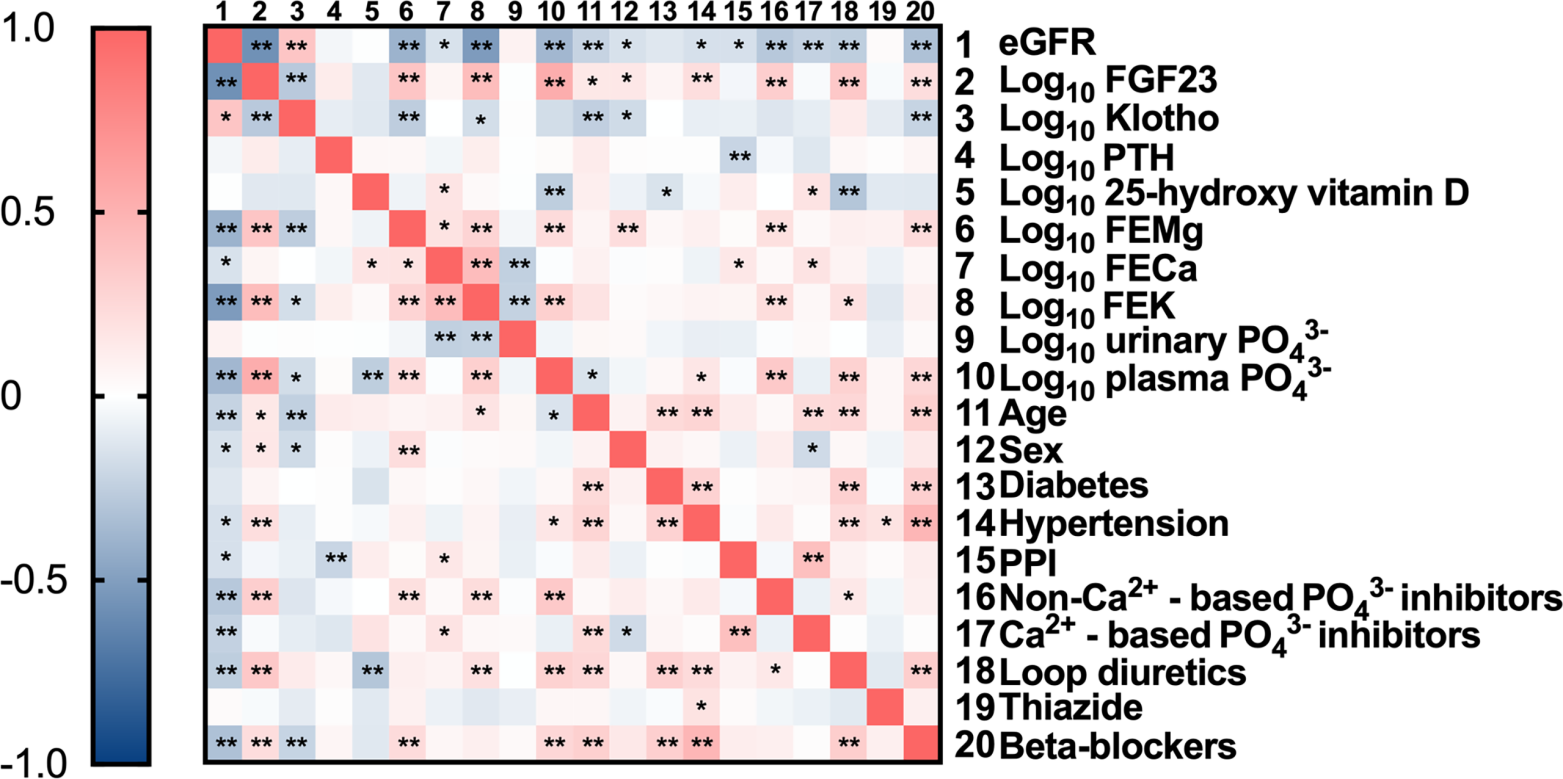
Correlation matrix with relevant parameters included. Data are the outcomes of bivariate analysis; *Correlation is significant at the 0.05 level; **Correlation is significant at the 0.01 level.

Univariate linear regression analysis revealed a significant correlation between plasma FGF23 and FEMg (in **Table 2**). Conversely, there was no significant correlation between FGF23 and plasma Mg^2+^ concentration and only a very weak correlation between FGF23 and urinary Mg^2+^ concentration (as seen in **Figure 2**).

**Table 2:** Linear regression models exploring the association between FGF23 and FEMg.

	Unstandardized β coefficient	95% CI	Adjusted R^2^	*p*-value
Crude	0.449	0.292 to 0.606	0.135	<0.001
Model 1	0.236	0.034 to 0.438	0.184	0.022
Model 2	0.204	-0.026 to 0.434	0.182	0.081
Model 3	0.269	0.032 to 0.506	0.181	0.026
Model 4	0.283	0.021 to 0.545	0.176	0.034
Model 5	0.295	0.029 to 0.562	0.167	0.030
Model 6	0.210	0.004 to 0.416	0.197	0.046
Model 7	0.189	-0.023 to 0.401	0.203	0.080

model 1 adjusted for age, sex and GFR Cystatin C.

model 2 adjusted for age, sex, GFR Cystatin C, log_10_ Klotho, log_10_ 25-hydroxyvitamin D, log_10_ PTH, log_10_ plasma PO_4_^3-^, log_10_ urinary PO_4_^3-^.

model 3 adjusted for age, sex, GFR Cystatin C, loop diuretics, thiazide, PPI, beta blockers, Ca^2+^-based PO_4_^3-^ binders, non-Ca^2+^-based PO_4_^3-^ binders.

model 4 adjusted for age, sex, GFR Cystatin C, log_10_ Klotho, log_10_ 25-hydroxyvitamin D, log_10_ PTH, log_10_ plasma PO_4_^3-^, log_10_ urinary PO_4_^3-^, loop diuretics, thiazide, PPI, beta blockers, Ca^2+^-based PO_4_^3-^ binders, non-Ca^2+^-based PO_4_^3-^ binders.

model 5 adjusted for age, sex, GFR Cystatin C, log_10_ Klotho, log_10_ 25-hydroxyvitamin D, log_10_ PTH, log_10_ plasma PO_4_^3-^, log_10_ urinary PO_4_^3-^, loop diuretics, thiazide, PPI, beta blockers, Ca^2+^-based PO_4_^3-^ binders, non-Ca^2+^-based PO_4_^3-^ binders, hypertension, diabetes.

model 6 adjusted for age, sex and GFR Cystatin C, log_10_ Klotho, log_10_ 25-hydroxyvitamin D, log_10_ PTH, log_10_FECa.

model 7 adjusted for age, sex, GFR Cystatin C, log_10_FECa, log_10_FEK, FEPi.

Next, we explored the relationship between FEMg and FGF23 after adjustment for potential confounders. In crude linear regression, FGF23 accounts for 37.4% of the variation in FEMg, with an adjusted R^2^ of 0.135 (p<0.001). After adjustment for age, sex and eGFR (model 1) there was an overall adjusted R^2^ of 0.184 (p=0.022). In a multiple adjusted linear regression model including age, sex, eGFR, Klotho, 25-hydroxyvitamin D, PTH, there was an overall adjusted R^2^ of 0.189, (p=0.059). In model 3 (adjusted for use of medication) there was an adjusted R^2^ of 0.179 (p=0.016), while model 4 showed an overall adjusted R^2^ of 0.174 (p=0.032). In the adjustment for medication and comorbidities (model 5) there was an adjusted R^2^ of 0.164 (p=0.029), while in the adjustment for fractional excretions (model 7) there was an adjusted R^2^ of 0.203 (p=0.080). The adjustment for the Klotho-FGF23 axis (model 6) showed an overall adjusted R^2^ of 0.197 (p=0.046). **Supplementary Tables 3, 4** show that the models in **Table 2** were not over-adjusted, using the variance inflation factor and two linear regression models.

### 3.4 Subgroup analysis by different eGFR and FGF23 stages

**Figure 3:**
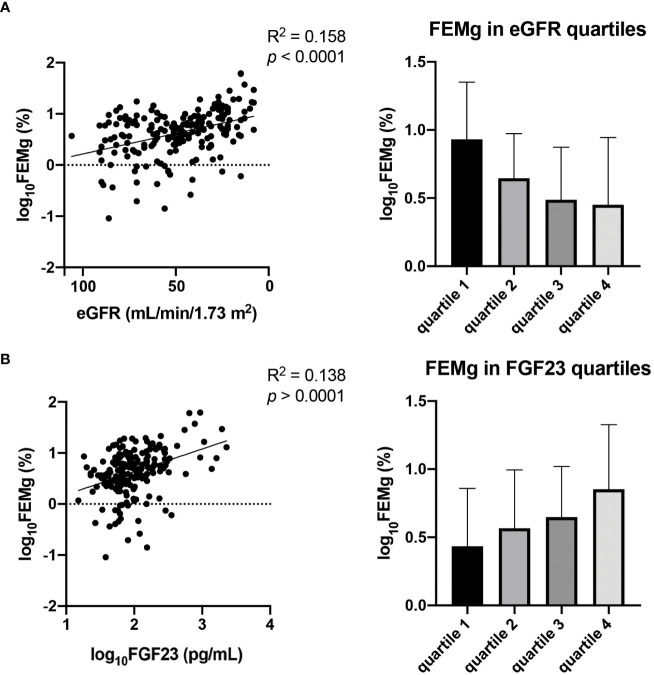
Association between eGFR and FEMg **(A)** in comparison with the association of FGF23 and FEMg **(B)**. Data are the outcomes of simple linear regression analysis.

To investigate if the associations to FEMg are consistent over the full spectra of FGF23 concentrations and eGFR observed in our cohort we performed sub-group analysis in the different strata of FGF23 and eGFR (shown in **Figure 3**). The results show that, in the FGF23 strata, the association between FEMg and FGF23 is significant only in the fourth quartile, while the association between FEMg and eGFR is significant in the first, third and fourth quartile. The division in eGFR quartiles demonstrated that FEMg correlates only in the first quartile with FGF23 (in **Table 3**). The pattern of FEMg is similar throughout FGF23 and eGFR strata – it decreases with the increase of eGFR and it simultaneously increases with FGF23 (**Figure 3**). **Supplementary Figures 2, 3** explore Klotho in CKD using similar analysis.

**Table 3:** Subgroup analysis of FEMg associated with FGF23 or eGFR in FGF23 or eGFR quartiles.

		FGF23		eGFR	
	Quartile range	R^2^	*p*-value	R^2^	*p*-value
FGF23 (pg/mL)					
quartile 1	15.1-57	0.013	0.932	-0.379	0.008
quartile 2	58-81	-0.162	0.252	-0.069	0.627
quartile 3	83-152	-0.035	0.810	-0.310	0.029
quartile 4	154-2307	0.340	0.018	-0.324	0.025
eGFR (mL/min/1.73 m^2^)					
quartile 1	8-30	0.300	0.035	-0.260	0.068
quartile 2	31-47	0.154	0.272	-0.233	0.093
quartile 3	48-66	0.155	0.310	-0.140	0.360
quartile 4	67-106	0.122	0.400	-0.162	0.262

## 4 Discussion

In this study of 198 non-dialysis CKD patients, we demonstrate that FGF23 is positively associated with FEMg independently of renal function and other potential confounders. This finding is consistent with the hypothesis that FGF23 may influence renal magnesium handling in patients with decreased renal function, and/or vice versa (23, 33, 34, 43).

The role of FGF23 in CKD and the associated cardiovascular disease has been the subject of many studies over the last decade (18, 39, 44, 45). As a master regulator of phosphate (46), it is well established that the increase in FGF23 contributes to maintaining phosphate homeostasis in early stages of CKD, and as such protects against vascular calcification. This is evidenced by studies (47) in which FGF23 neutralizing antibodies were given to rats with CKD, where the reduction of FGF23 resulted in hyperphosphatemia and significantly worsen vascular calcification. However, it is still debated whether the dramatically increased FGF23 concentrations commonly observed in late-stage CKD patients have direct and detrimental effects on the cardiovascular system.

Similarly, the role of Mg^2+^ homeostasis in CKD patients has received increasing attention over the last few years, especially in the context of vascular calcification and other cardiovascular morbidities (17, 20, 48). There have been some indications that Mg^2+^ is involved in controlling FGF23 levels (38, 39), and that FGF23-Klotho signaling might influence renal magnesium handling. A potential mechanism for such relationship might be that FGF23-Klotho signaling in the DCT controls TRPM6 expression and/or activity. This is supported by the co-expression of Klotho and TRPM6 in distal tubular cells (49, 50), and by the fact that the renal expression of Klotho and TRPM6 are strongly correlated in healthy individuals as well as in patients with diabetic nephropathy (51).

Although a few studies have reported on associations between FGF23 and Mg^2+^ in animals or in patients with ESRD, no studies have explored their potential relationship in human non-dialysis CKD (40, 41). In the present study we report a direct and independent association between FGF23 and FEMg, whereas there are no or only weak associations to plasma or urinary Mg^2+^. This is supportive of the hypothesis that FGF23 may participate in controlling renal Mg^2+^ reabsorption, to maintain Mg^2+^ homeostasis as renal function declines (23, 33, 34, 43). The plasma Mg^2+^ levels were stable across all quartiles, which indicates a sustained intestinal absorption of Mg^2+^ due to dietary Mg^2+^ intake (4, 52). However, the cross-sectional nature of this study restricts us from drawing conclusions about any possible causality behind the observed relationship between FEMg and FGF23.

Importantly, as the hormonal regulation of Mg^2+^ handling is incompletely understood, there could be confounders that we did not consider in our models and that might explain the observed relationship, which could provide a potential mechanism and explanation to the observed associations.

In conclusion, our study demonstrates that FGF23 is positively associated with FEMg in patients with CKD, independently of renal function and other potential confounders. These finding warrants further investigations of the FGF23-Klotho-Pi-Mg^2+^ axis in patients with CKD and vascular calcification.

## Data availability statement

The data that support the findings of this study are not publicly available due to their containing information that could compromise the privacy of research participants, but are available from KaroKidney upon reasonable request.

## Ethics statement

The study received ethical approval from the Ethical Review Board, Stockholm, Sweden (DNR 2010/579-31), and informed consent was obtained from all participants.

## Author contributions

TG, HO, and JH were involved in the conception and design of the study. TG performed experiments and statistical analysis. AWi, PB, AWe, AB and HO created the biobank. TG, MZ, HO and JH drafted the manuscript. TG, MZ, HX, AWi, PB, AWe, AB, HO and JH critically revised the manuscript. TG, MZ, AWi, PB, AWe, AB, HX, HO and JH approved the final version of the manuscript. All authors contributed to the article and approved the submitted version.
