# Supplementary material for: Fibroblast growth factor 23 is independently associated with renal magnesium handling in patients with chronic kidney disease
Source: Front Endocrinol (Lausanne). 2023 Jan 9;13:1046392. doi: 10.3389/fendo.2022.1046392 (PMC9869122; doi:10.3389/fendo.2022.1046392)
Supplement: Supplementary file 1 [file DataSheet_1.docx]

Supplementary Material

# Supplementary Figures and Tables

## Supplementary Figures


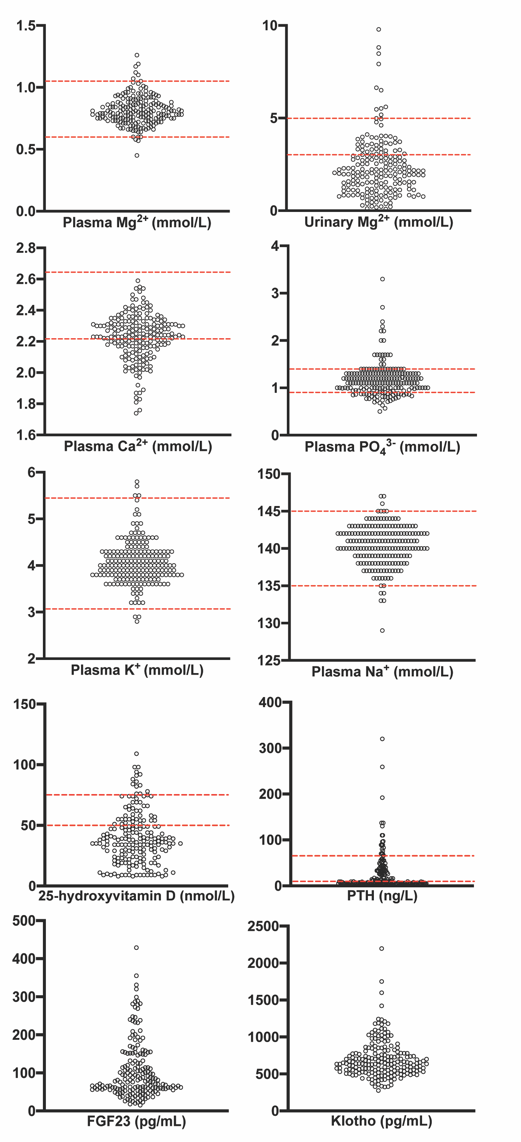


**Supplementary Figure 1.** Graphical representation of electrolytes, 25-hydroxyvitamin D, PTH, FGF23 and Klotho distribution. Red lines represent the normal levels.


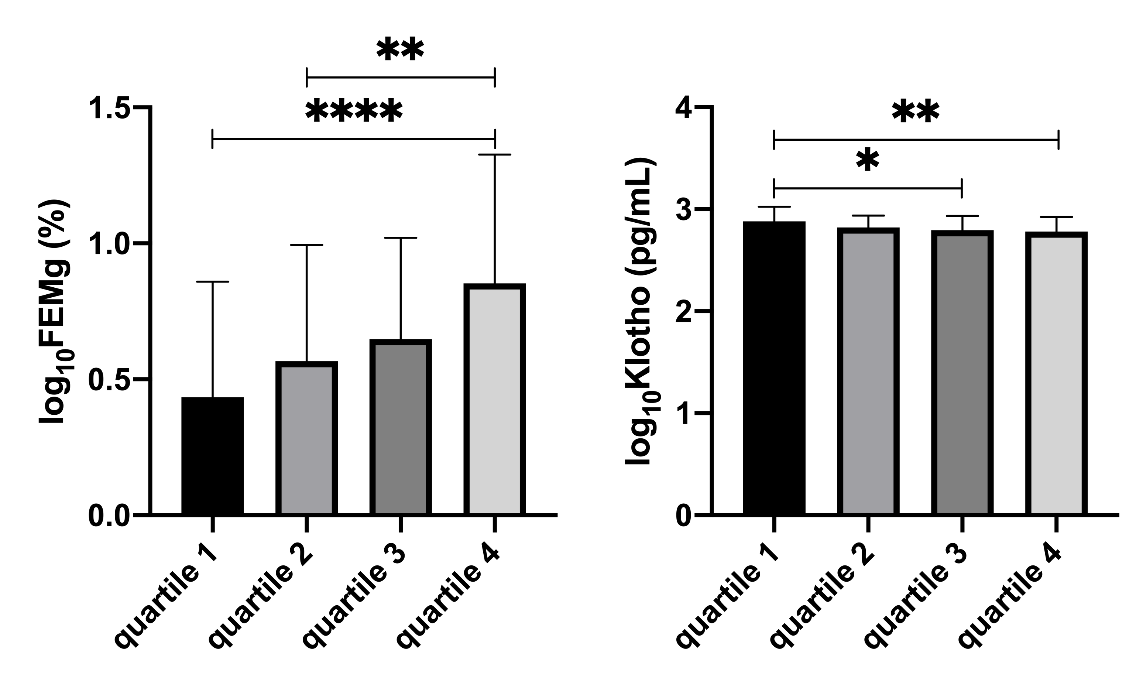


**Supplementary Figure 2.** FGF23 quartile group comparison of FEMg and Klotho.


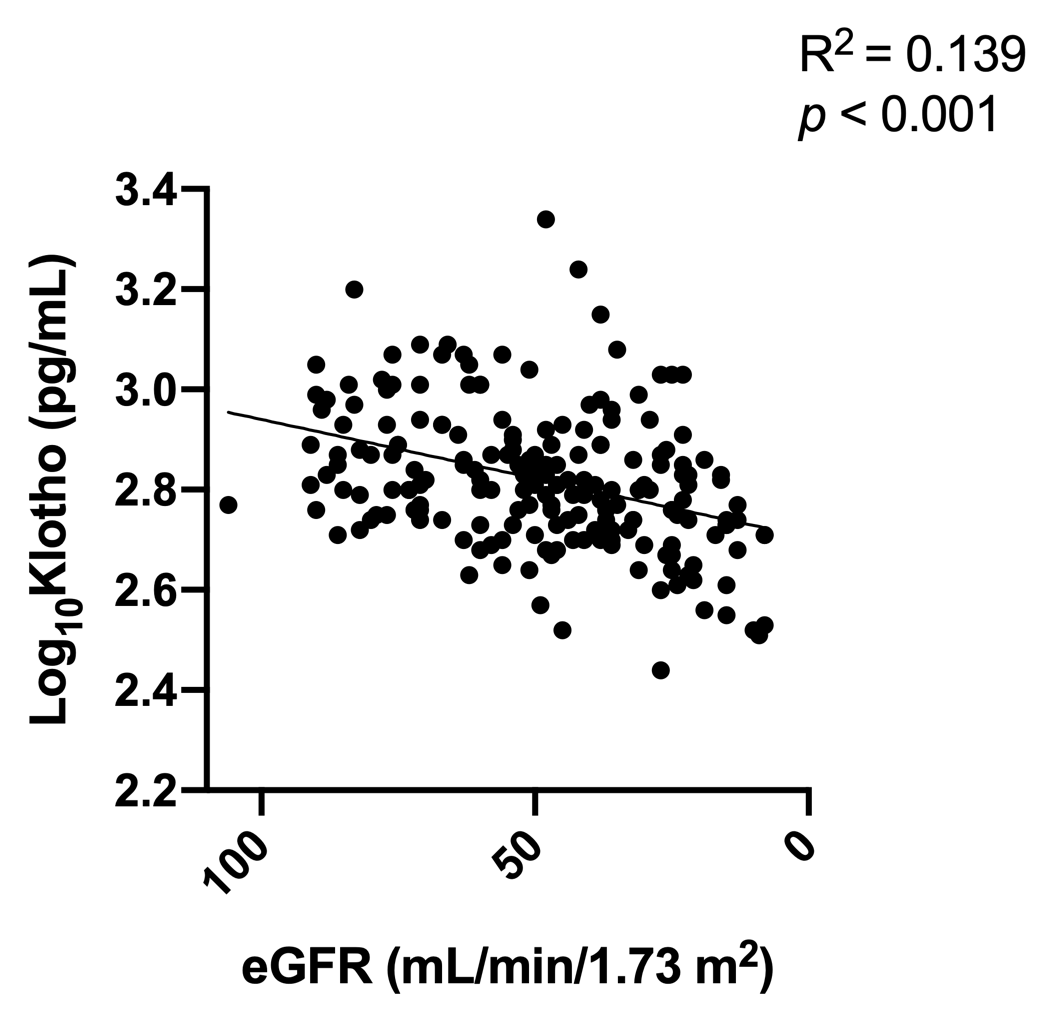


**Supplementary Figure 3.** Association between log_10_Klotho and eGFR.

## Supplementary Tables

| Variable | Missing percentage (%) |
| --- | --- |
| FGF23 (pg/mL) | 0.51 |
| GFR Cystatin C (mL/min/1.73 m^2^) | 12.63 |
| Aldosterone (pg/mL) | 8.08 |
| PTH (ng/L) | 14.14 |
| 25-hydroxyvitamin D (nmol/L) | 4.55 |
| Urinary Ca^2+^ (mmol/L) | 14.14 |
| FECa (%) | 8.08 |
| Urinary PO_4_^3-^ (mmol/L) | 14.14 |
| FEPi (%) | 8.08 |
| FEK (%) | 8.08 |
| Urinary Na^+^ (mmol/L) | 15.66 |
| Urinary Cl^-^ (mmol/L) | 12.12 |

**Supplementary Table 1.** Missing data, 198 cases

|  | Log_10_FEMg | | Log_10_FGF23 | | Log_10_Klotho | |
| --- | --- | --- | --- | --- | --- | --- |
|  | Pearson’s correlation coefficient | *p*-value | Pearson’s correlation coefficient | *p*-value | Pearson’s correlation coefficient | *p*-value |
| Age  (years) | 0.068 | 0.338 | 0.150 | 0.035 | -0.248 | <0.001 |
| Sex  (%) | 0.220 | 0.003 | 0.151 | 0.044 | -0.192 | 0.010 |
| Log_10_FGF23  (pg/mL) | 0.374 | <0.001 | N/A | N/A | -0.282 | <0.001 |
| GFR Cystatin C  (mL/min/1.73 m^2^) | -0.397 | <0.001 | -0.577 | <0.001 | 0.373 | <0.001 |
| Log_10_Klotho  (pg/mL) | -0.261 | <0.001 | -0.282 | <0.001 | N/A | N/A |
| Log_10_PTH  (ng/L) | 0.050 | 0.487 | 0.124 | 0.082 | -0.097 | 0.172 |
| Log_10_25-hydroxyvitamin D (nmol/L) | -0.063 | 0.376 | -0.121 | 0.088 | -0.123 | 0.083 |
| Plasma Mg^2+^  (mmol/L) | -0.019 | 0.790 | 0.017 | 0.811 | -0.035 | 0.620 |
| Log_10_Urinary Mg^2+^  (mmol/L) | 0.555 | <0.001 | -0.146 | 0.040 | 0.059 | 0.411 |
| Log10FEMg  (%) | N/A | N/A | 0.374 | <0.001 | -0.261 | <0.001 |
| Log_10_FECa  (%) | 0.183 | 0.010 | 0.065 | 0.365 | -0.001 | 0.989 |
| Log_10_Plasma PO_4_^3-^  (mmol/L) | 0.229 | 0.001 | 0.532 | <0.001 | -0.182 | 0.010 |
| Log_10_Urinary PO_4_^3-^  (mmol/L) | -0.048 | 0.502 | -0.010 | 0.894 | 0.010 | 0.885 |
| FEPi  (%) | 0.394 | <0.001 | 0.527 | <0.001 | -0.344 | <0.001 |
| Log_10_FEK  (%) | 0.275 | <0.001 | 0.429 | <0.001 | -0.176 | 0.013 |
| Diabetes | 0.047 | 0.512 | 0.069 | 0.333 | 0.000 | 0.996 |
| Hypertension | 0.096 | 0.176 | 0.213 | 0.003 | -0.090 | 0.207 |
| PPI | 0.026 | 0.727 | -0.047 | 0.522 | -0.082 | 0.268 |
| Non-Ca^2+^-based PO_4_^3-^ binders | 0.202 | 0.006 | 0.318 | <0.001 | -0.136 | 0.065 |
| Ca^2+^-based PO_4_^3-^ binders | 0.044 | 0.550 | -0.028 | 0.703 | -0.098 | 0.186 |
| Loop diuretics | -0.300 | <0.001 | 0.353 | <0.001 | 0.129 | 0.080 |
| Thiazide | -0.118 | 0.112 | -0.030 | 0.686 | -0.105 | 0.156 |
| Beta-blockers | -0.124 | 0.095 | 0.220 | 0.003 | -0.227 | 0.002 |

**Supplementary Table 2.** Bivariate analysis for correlation with log_10_FEMg, log_10_FGF23, and log_10_Klotho.

| **Model** | **Collinearity** | |
| --- | --- | --- |
|  | **Variable** | **VIF** |
| Crude | - | - |
| 1 | Log_10_FGF23 | 1.539 |
|  | Age | 1.070 |
|  | Sex | 1.033 |
|  | GFR Cystatin C | 1.599 |
| 2 | Log_10_FGF23 | 1.625 |
|  | Age | 1.120 |
|  | Sex | 1.058 |
|  | GFR Cystatin C | 1.746 |
|  | Log_10_Klotho | 1.293 |
|  | Log_10_vitamin D | 1.071 |
|  | Log_10_PTH | 1.043 |
|  | Log_10_ Plasma PO_4_^3-^ | 1.672 |
|  | Log_10_ Urinary PO_4_^3-^ | 1.025 |
| 3 | Log_10_FGF23 | 1.791 |
|  | Age | 1.193 |
|  | Sex | 1.056 |
|  | GFR Cystatin C | 1.780 |
|  | PPI | 1.089 |
|  | Beta blockers | 1.312 |
|  | Loop diuretics | 1.327 |
|  | Thiazides | 1.038 |
|  | Ca^2+^-based PO_4_^3-^ binders | 1.510 |
|  | Non-Ca^2+^-based PO_4_^3-^ binders | 1.195 |
| 4 | Log_10_FGF23 | 1.858 |
|  | Age | 1.279 |
|  | Sex | 1.103 |
|  | GFR Cystatin C | 1.926 |
|  | PPI | 1.173 |
|  | Beta blockers | 1.361 |
|  | Loop diuretics | 1.589 |
|  | Thiazides | 1.095 |
|  | Log_10_PTH | 1.124 |
|  | Log_10_Klotho | 1.425 |
|  | Log_10_vitamin D | 1.274 |
|  | Log_10_ Plasma PO_4_^3-^ | 1.975 |
|  | Log_10_ Urinary PO_4_^3-^ | 1.036 |
|  | Ca^2+^-based PO_4_^3-^ binders | 1.532 |
|  | Non-Ca^2+^-based PO_4_^3-^ binders | 1.307 |
| 5 | Log_10_FGF23 | 1.928 |
|  | Age | 1.385 |
|  | Sex | 1.106 |
|  | GFR Cystatin C | 1.937 |
|  | PPI | 1.189 |
|  | Beta blockers | 1.651 |
|  | Loop diuretics | 1.620 |
|  | Thiazides | 1.161 |
|  | Log_10_PTH | 1.144 |
|  | Log_10_Klotho | 1.435 |
|  | Log_10_vitamin D | 1.285 |
|  | Hypertension | 1.479 |
|  | Diabetes | 1.265 |
|  | Log_10_ Plasma PO_4_^3-^ | 1.981 |
|  | Log_10_ Urinary PO_4_^3-^ | 1.048 |
|  | Ca^2+^-based PO_4_^3-^ binders | 1.537 |
|  | Non-Ca^2+^-based PO_4_^3-^ binders | 1.311 |
| 6 | Log_10_FGF23 | 1.627 |
|  | Age | 1.126 |
|  | Sex | 1.060 |
|  | GFR Cystatin C | 1.760 |
|  | Log_10_PTH | 1.040 |
|  | Log_10_Klotho | 1.296 |
|  | Log_10_vitamin D | 1.104 |
|  | Log_10_FECa | 1.061 |
| 7 | Log_10_FGF23 | 1.727 |
|  | Age | 1.105 |
|  | Sex | 1.053 |
|  | GFR Cystatin C | 2.014 |
|  | Log_10_FECa | 1.290 |
|  | Log_10_FEK | 1.820 |
|  | FEPi | 2.112 |

**Supplementary Table 3.** Variance inflation factor for variables included in the regression models from Table 2.

| **Model** | **Unstandardized β coefficient** | **95% CI** | **Adjusted R^2^** | ***p*-value** | **Collinearity** | |
| --- | --- | --- | --- | --- | --- | --- |
|  |  |  |  |  | **Variable** | **VIF** |
| a | 0.381 | 0.218 to 0.544 | 0.155 | <0.001 | Log_10_FGF23 | 1.113 |
|  |  |  |  |  | Log_10_hydroxyvitamin D | 1.041 |
|  |  |  |  |  | Log_10_Klotho | 1.115 |
| b | 0.442 | 0.284 to 0.601 | 0.131 | <0.001 | Log_10_FGF23 | 1.014 |
|  |  |  |  |  | Log_10_hydroxyvitamin D | 1.014 |

**Supplementary Table 4.** Linear regression models exploring the possible over-adjustment for Klotho in the association between FGF23 and FEMg.

*model a corrected for Log_10_Klotho and Log_10_hydroxyvitamin D

model b corrected for Log_10_hydroxyvitamin D
